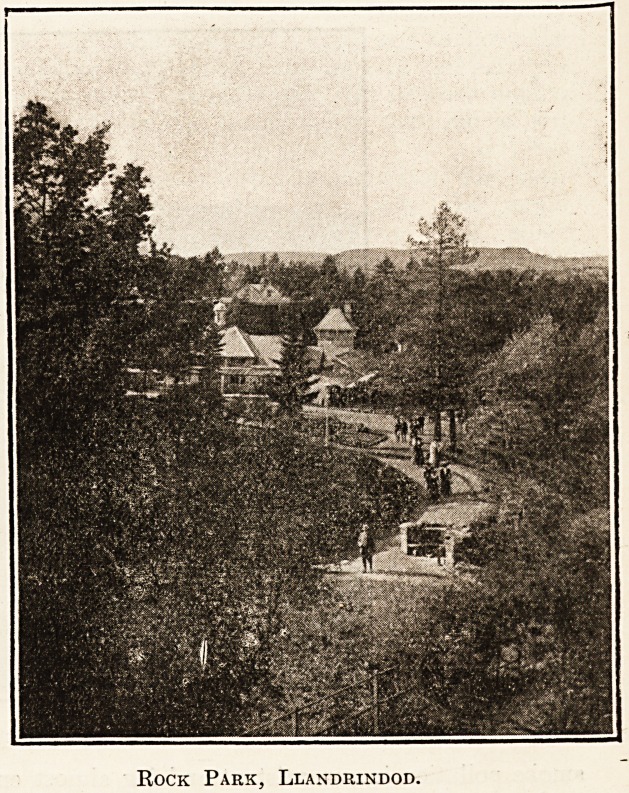# Home and Foreign Spas
*Previous articles in this series appeared in The Hospital of Jan. 28, Feb. 25, March 25, April 22 and May 20.


**Published:** 1911-06-03

**Authors:** 


### HOME AND FOREIGN SPAS.*

### VI. LLANDRINDOD WELLS.

Wales is renowned for its great natural beauty, and the country surrounding Llandrindod Wells is no exception to the rule which so generally prevails throughout the Principality. The Spa stands on a plateau amid the Radnorshire Hills, some 700 to 800 feet above sea-level, and as a health-resort it possesses, by virtue of its exceptionally invigorating climate, as well as its numerous medicinal springs, a reputation which few places in this country or the Continent, can more justly claim. The town is quite modern and well laid out; there are no factories, public works, or industrial centres in the neighbourhood, consequently smoke pollution is non-existent and fog almost entirely absent. On the west side there is nothing but hills between the Spa and the Atlantic Ocean, some forty miles away, which, no doubt, is partly responsible for the tonic and bracing influence of the air and the singularly fresh cooling breezes that are common in summer. Eastward is the Radnor Forest range of hills, about 2,000 feet high, forming an excellent screen from wind in that cold quarter. The temperature is equable, and the atmosphere comparatively dry, the average rainfall for the past ten years being 37 inches, which is below that for the West of England. A notable feature of the Spa is the rapidity with which the ground dries after rain; the elevation and general conformation of the land help to carry off the surface water, and in a short time the roads are remarkably clean and dry.

The domestic water-supply is abundant and of excellent quality, while the sewerage system is perfect; septic tank and filter beds are employed with most satisfactory results. As evidence of the healthiness of the town, it is worthy of note that infectious diseases are practically unknown, and the average death rate for the last ten years is 5.5 per 1,000 inhabitants.

Llandrindod Wells is 214 miles from London (Euston), and is reached within 5¾ hours by the London and North Western Railway Company's through service. It is also easily accessible from the chief provincial centres such as Birmingham, Manchester, and Liverpool, and as most of the trains are equipped with corridor carriages and restaurant cars, the journey can be performed without trouble or inconvenience.

General View of Llandrindod Wells.

### The Springs, their Nature and Constituents.

Owing to the geological formation of the district, the Spa is peculiarly rich in medicinal mineral waters, all of which contain a proportion of chlorides of sodium, calcium, and magnesium, and are divided into the following classes: Simple saline, lithia saline, magnesium, muriated sulphur and chalybeate. The two saline waters are similar to those of Homburg and Kissengen, and are usually taken warm in considerable quantities before breakfast, although this depends largely upon the patient and the disease for which they are prescribed. Their action is gentle, and they are especially indicated in the treatment of all rheumatic and gouty affections. As an active agent in the relief of many dyspeptic conditions, the magnesium water, which is also a weak saline, containing a considerable

* Previous articles in this series appeared in The Hospital of Jan. 28, Feb. 25, March 25, April 22 and May 20.

proportion of magnesium chloride, has proved to be exceptionally efficacious. The sulphur waters, of which there are several, vary in strength and composition, and their therapeutic value is too well appreciated by the medical profession to need comment here. A glance at the analysis of the radium sulphur and lithia saline springs will give an idea of the valuable nature of these two springs in particular. There are two chalybeate springs containing a small quantity of bicarbonate of iron, and these have been found useful in the treatment of anæmia and other debilitated conditions. The following is a comparative analyses of the various springs:—

| | *Mineral constituents in grains and gases in cubic inches per imperial gallon.* | | | | |
|---|---|---|---|---|---|
| Name of Spring ... | Radium Red Sulphur Spring | Chalybeate Spring | New Spring | Magnesium Spring | Lithia Saline Springs |
| Name of Spa ... | Park Spa | Park Spa | Recr'tn Ground | Park Spa | Park Spa |
| Name of Analyst ... ... | Embrey | Swete | Thresh | Swete | Embrey |
| Date ... ... ... | 19C4 | 1879 | 1907 | 1894 | 1906 |
| Chloride of Sodium ... | 80.7 | 278.30 | 163.6 | 236.46 | 279.8 |
| Chloride of Calcium ... | 30.8 | 64.73 | 110.9 | 88.9 | 73.26 |
| Chloride of Magnesia ... | 14.34 | 13.75 | 37.7 | 49.42 | 14.01 |
| Chloride of Potassium ... | 0.93 | 1.21 | ... | 1.4 | ... |
| Chloride of *Lithium* ... | 0.34 | faint trace | ... | trace | 3.83 |
| Carbonate of *Lithium* ... | ... | ... | ... | ... | 5.7 |
| Chloride of Thallium ... | ... | ... | ... | trace | 1.2 |
| Carbonate of Calcium ... | 1.6 | 0.61 | 10.3 | ... | ... |
| Carbonate of Ammonia ... | ... | 0.14 | ... | 0.19 | ... |
| Silica ... ... ... | 0.82 | 1.33 | trace | 4.14 | ... |
| Nitrate of Calcium ... | ... | 0.01 | ... | ... | ... |
| Sulphate of Calcium ... | ... | 0.71 | 6.8 | ... | ... |
| Iron Oxide ... ... | 0.41 | ... | ... | 0.7 | trace |
| Oxide of Aluminium ... | 0.3 | ... | ... | 1.05 | 3.34 |
| Carbonate of Magnesia ... | 2.49 | ... | ... | ... | ... |
| Carbonate of Iron ... | ... | 1.26 | 1.6 | ... | ... |
| Bromide of Potassium ... | ... | a trace | ... | ... | ... |
| Iodide of Potassium ... | ... | a trace | ... | ... | ... |
| Nitrate of Potash ... | ... | ... | ... | ... | ... |
| Nitrates ... ... ... | ... | ... | ... | traces | ... |
| Nitrities ... ... ... | ... | ... | ... | nil | ... |
| Bromide of Sodium ... | ... | ... | 0.6 | ... | ... |
| Iodide of Sodium ... | ... | ... | trace | ... | ... |
| Alumina ... ... ... | ... | ... | trace | ... | ... |
| Water of Hydration ... | ... | ... | 13.6 | ... | ... |
| Radium ... ... ... | strong trace | ... | ... | ... | ... |
| Nitrogen ... ... ... | 14.2 | ... | ... | 0.28 | 1.0 |
| Oxygen ... ... ... | 8.1 | ... | ... | 0.23 | 0.5 |
| Sulphuretted Hydrogen . | 11.35 | ... | ... | 0.80 | ... |
| Carbonate Acid ... ... | 2.2 | ... | ... | 1.60 | 2.5 |

### DISEASES TREATED.

Medically prescribed or externally applied in one or other of the various forms of treatment, the waters of Llandrindod have proved, in conjunction with the climate, to be of considerable assistance in the alleviation of gout, rheumatism, rheumatoid arthritis, liver complaints, neuritis, sciatica, lumbago, neurasthenia, anæmia, gravel, and renal calculi, colitis, diabetes and glycosuria, chronic skin diseases and catarrhal conditions of the throat and nose.

### THE PUMP ROOMS.

There are two pump rooms; the Rock Park Spa, and the old Pump House, adjoining the Pump House Hotel. Of these, the former is by far the more popular; it is situate in the Rock Spa Park, the source of the more important mineral springs in Llandrindod, a veritable beauty spot, with scenery almost alpine in its character. Here the waters are dispensed throughout the day from early morning, at any temperature that may be prescribed, and it undoubtedly adds greatly to the value of the "cure" when taken amid such delightful surroundings. There are plenty of comfortable seats thoughtfully placed in the most shady corners, and as excellent music is provided a most enjoyable time may be spent on a summer's day in this charming park.

### THE BATHING ESTABLISHMENTS.

In the spring of 1909 several enterprising residents saw the necessity of installing a thoroughly up-to-date system of medicinal and electric baths,

ROCK PARK, LLANDRINDOD.

and with this object, suitable and commodious premises were secured in a central position in the High Street, within a few minutes' walk of the railway station, principal hotels, boarding houses and pump rooms; in fact, a more favourable situation it would indeed be difficult to find. The installation includes the most approved appliances, and provides all treatments known to modern hydrotherapy, namely, Vichy, Aix, and Scotch douches, Plombiéres treatment with Tivoli external douche, needle, peat, mud and electric light baths, arc light, Dr. Tyrnauer's hot air apparatus, and various hydro electric and medicated baths, such as pine, brine, sulphur, coal-tar, etc. There is also a simple arrangement for giving carbonic acid baths, and an excellent $x$-ray department for diagnosis and treatment, an acquisition which has proved of inestimable service. The management of this establishment and the Rock Park Spa is in the capable hands of Mr. George Baillie, at one time experimental electrical engineer to Lord Kelvin, and it is due to Mr. Baillie's knowledge and foresight that all details of heating, ventilating, and the electrical installation has been so carefully carried out. It is these small things, so often overlooked, that add so greatly to the comfort of the patients. In this connection there are two features worthy of particular remark; one is the almost entire absence of steam in the baths, no matter at what temperature the water may be, this is due to an ingenious arrangement of fans, and is unquestionably a move in the right direction, as in many baths it is almost impossible to breathe properly in consequence of the excess of steam in the atmosphere; the other is the employment of a super floor of wood laths over the usual tile or stone floor, this is a distinct comfort, especially to rheumatic patients, to whom the chill of a tile floor is often so objectionable, if not dangerous. In addition to the suites of baths, the establishment contains a luxurious lounge and waiting rooms, tastefully decorated in green and white, a colour scheme which prevails throughout the building. The Rock Spa Park Baths, in the Rock Park, have recently come under the same management as the High Street Baths, and will shortly be completely overhauled and equipped in a thoroughly up-to-date manner.

### Accommodation.

There is a fair number of well-appointed hotels and boarding houses, of which, perhaps, the Bridge Hotel is now the largest. Every season some enlargement of the premises is necessary in order to cope with the increasing number of its patrons. As the town is essentially a Spa, and relies entirely upon visitors for its support, it is possible to secure comfortable apartments in close proximity to the Pump Rooms and bathing establishments at really reasonable charges, and for those patients suffering from nervous disorders, a private establishment is to be preferred to the more active life which cannot be avoided in large hotels and boarding houses.

### Amusements and Recreations.

As the season is essentially a summer one, the question of outdoor recreation only need concern us here. Abundant provision is made for lawn tennis, bowling, croquet, quoits, and golf; many of the hotels and boarding houses have their own tennis lawns and bowling greens, whilst new tennis lawns and a bowling green have been laid out in the Recreation Ground, which are available to visitors on payment of a nominal charge. The beautiful ornamental lake adjoining the Common affords excellent facilities for boating, and fishing may be obtained in the Ithon and its tributary streams. There are many places of interest in the vicinity, to which motor brakes and cars run daily during the season, among which may be mentioned the beautiful Elan Valley, containing Abbey-Cwinher, the Birmingham Corporation reservoir, and three large dams, one of the most remarkable of engineering feats of recent years.

## Figures and Tables

**Figure f1:**
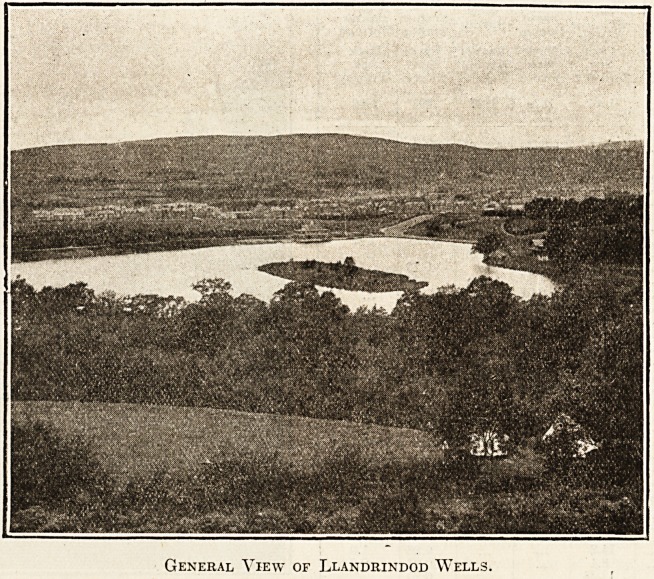


**Figure f2:**